# A comprehensive list of human microdeletion and microduplication syndromes

**DOI:** 10.1186/s12863-022-01093-3

**Published:** 2022-11-26

**Authors:** Alyssa S. Wetzel, Benjamin W. Darbro

**Affiliations:** Stead Family Department of Pediatrics, University of Iowa Hospitals and Clinics, Iowa City, IA 52242 USA

**Keywords:** Copy number variant, Microdeletion, Microduplication, Microduplication/microdeletion disorders, Genomic disorders

## Abstract

**Objective:**

The phenotypic spectrum of human microdeletion and microduplication syndromes (MMS) is heterogeneous but often involves intellectual disability, autism spectrum disorders, dysmorphic features and/or multiple congenital anomalies. While the common recurrent copy number variants (CNVs) which underlie these MMS have been well-studied, the expansion of clinical genomic testing has led to the identification of many rare non-recurrent MMS. To date, hundreds of unique MMS have been reported in the medical literature, and no single resource exists which compiles all these MMS in one location. This comprehensive list of MMS will aid further study of CNV disorders as well as serve as a resource for clinical laboratories performing diagnostic CNV testing.

**Data description:**

Here we provide a comprehensive list of MMS which have been reported in the medical literature to date. This list is sorted by genomic location, and for each MMS, we provide a list of publications for referral, as well as the consensus coordinates, representative region, shortest regions of overlap (SRO), and/or subregions where applicable.

## Objective

While the field of genetics is over 150 years old, it has only been within the last twenty years that we have begun to understand the role of copy number variants (CNVs) in human disease [1]. In the early 1990s, the underlying microdeletion was identified for several clinical syndromes [2–6]. These discoveries led to research on the surrounding genomic architecture and mechanism of formation for these microdeletions which in turn led to the discovery of the predicted reciprocal microduplications and their associated clinical syndromes. Then, the completion of the human genome project and development of more cost-effective sequencing technologies in the early 2000s paved the way for the expansion of the genotype-first approach to identifying genomic disorders [2, 3, 7]. The spectrum of CNVs which have been identified has been further expanded since the American College of Medical Genetics and Genomics formally recommended the chromosomal microarray as a first-line diagnostic test. Cooper et al. [8] leveraged both patient and control population data to identify regions of the human genome which are enriched for CNVs in patient population identifying 59 putative pathogenic regions. While 14 of these putative regions were novel and/or weakly supported at the time, several have since been decidedly associated with human disorders. In 2014, Nevado et al. [9] reported that nearly 100 *new* CNV disorders had been reported in a five-year period, further underscoring the breadth and depth of the role of CNVs in human disease. Unlike prior reviews of microdeletion and microduplication syndromes (MMS) [9–11], this comprehensive list provides both genomic coordinates and a list of representative publications for phenotypic analysis and/or further reading. Thus, this list serves as a valuable resource in the interpretation of clinically identified CNVs or for use in downstream variant prioritization or classification pipelines.

## Data description

A non-redundant list of microduplication and microdeletion syndromes (MMS) was obtained from the following databases and publications: Clinical Genome Resource (ClinGen; www.clinicalgenome.org) [12] via UCSC’s Table Browser (iscaCuratedPathogenic; *n* = 61) [13, 14], DatabasE of genomiC varIation and Phenotype in Humans using Ensembl Resources (DECIPHER; https://decipher.sanger.ac.uk/;*n* = 66) [15], Online Mendelian Inheritance in Man (OMIM; www.omim.org) [16] via the Gene2Map file (keywords: contiguous gene, chromosome (exclude: open reading frame), deletion, duplication, triplication, and quadruplication; *n* = 115), Nevado et al. [9] (*n* = 99), Table 2 of Bentacur [10] (*n* = 39), Supplemental Tables 1 and 2 of Kaminsky et al. [17] (*n* = 41), Additional File 5 of Marcinkowska et al. [18] (*n* = 20) and Table 1 of Wiese et al. [11] (*n* = 193).

The non-redundant list was curated to exclude disorders which were only described in a single patient (e.g. 3p11.2-p12.1 [9, 19]) or family and disorders which are not asssociated with congenital disease (e.g. 8p11 myeloproliferative syndrome; OMIM 613523). CNV disorders were retained even if only the duplication or deletion has been observed or assoicated with human disease. When possible, coordinates for the CNV disorders were obtained from either DECIPHER or ClinGen (accessed August 2021). For all remaining MMS without consensus coordinates, CNV coordinates for each reported patient were identified as were the coordinates for any defined shortest region of overlaps (SRO) or minimal regions (Dataset 2). All coordinates were converted from their initial assembly into hg19 coordinates using UCSC’s Liftover Tool [20]. These patient and minimal regions were utilized to define the representative CNV interval and when applicable minimal region(s) of overlap. Some MMS were divided into multiple interval regions to account for: recurrent CNVs with multiple breakpoints (e.g. 15q11.2 BP1–2 vs 15q11.2 BP1–3 vs 15q11.2 BP2–3), size differences due to the presence of segmental duplications within a CNV call (1q21.1 TAR Susceptibility Locus), and to account for multiple minimal CNV regions (e.g. 1q24-q25). Further details on the representative and minimal intervals for each CNV disorder can be found in Datafile 1. BED file versions of Dataset 1 are available for hg19 (Dataset 3) and GRCh38 (Dataset 4) coordinates.

We identified 192 recurrent and non-recurrent Microdeletion and Microduplication Syndromes (Dataset 1) which were divided up into 320 individual CNV intervals and 141 non-overlapping CNV regions. Of these intervals, 25% (*n* = 80) had consensus coordinates from ClinGen and/or DECIPHER. Coordinates for the remaining CNV intervals (*n* = 240) were determined manually by leveraging over 2500 patient CNVs, defined SRO(s) or minimal region(s) from the medical literature (Datafile 1; Dataset 2).

**Table:** 

Label	Name of Data Set/File	File Type/Extension	Data repository and identifier (DOI or accession number)
***Dataset 1***	*Microdeletion and Microduplication Syndromes*	*MS Excel file (.xlsx)*	Zenodo (10.5281/zenodo.6975072) [21]
***Dataset 2***	*MMS CNVs Reported in Medical Literature*	*MS Excel file (.xlsx)*	Zenodo (10.5281/zenodo.6975072) [21]
***Dataset 3***	*MMS hg19*	*Browser Extensible Data (.bed)*	Zenodo (10.5281/zenodo.6975072) [21]
***Dataset 4***	*MMS GRCh38*	*Browser Extensible Data (.bed)*	Zenodo (10.5281/zenodo.6975072) [21]
***Datafile 1***	*CNV Coordinate Determination by Chromosome*	*MS Word file (.doc)*	Zenodo (10.5281/zenodo.6975072) [21]
***Datafile 2***	*Reference List for Microdeletion and Microduplication Syndromes*	*MS Word file (.doc)*	Zenodo (10.5281/zenodo.6975072) [21]

## Limitations

We chose to limit the scope of this MMS list to the characterization of the representative genomic coordinates rather than conducting a thorough phenotypic review of each reported MMS. Therefore, researchers and clinicans will need to refer to each MMS’ reference list or other databases such as OMIM to collect this information. This list of MMS was restricted to those described in at least two unrelated patients, and relied on databases and prior reveiews of MMS. Therefore, there is the potential that a MMS has been missed and a future iteration(s) of this comprehenisve MMS list will likely be needed.

## Data Availability

The data described in Table 1 of this Data Note can be freely and openly accessed on GitHub under aswetzel/MMS (https://github.com/aswetzel/MMS) [21].

## Abbreviations

CNVs: Copy number variants
MMS: Microdeletion and microduplication syndromes
ClinGen: Clinical Genome Resource
DECIPHER: DatabasE of genomiC varIation and Phenotype in Humans using Ensembl Resources
OMIM: Online Mendelian in Man
SRO: Shortest region of overlap
